# Slot-Die Coating of Double Polymer Layers for the Fabrication of Organic Light Emitting Diodes

**DOI:** 10.3390/mi10010053

**Published:** 2019-01-14

**Authors:** Amruth C, Marco Colella, Jonathan Griffin, James Kingsley, Nicholas Scarratt, Beata Luszczynska, Jacek Ulanski

**Affiliations:** 1 Department of Molecular Physics, Lodz University of Technology, Lodz 90 924, Poland; amruth.c@p.lodz.pl (A.C.); jacek.ulanski@p.lodz.pl (J.U.); 2 Department of Physics, Durham University, Durham DH1 3LE, UK; marco.colella@durham.ac.uk; 3 Ossila Limited, Sheffield S4 7WB, UK; j.griffin@ossila.com (J.G.); j.kingsley@ossila.com (J.K.); n.scarratt@ossila.com (N.S.)

**Keywords:** slot-die coating, organic light emitting diodes (OLEDs), printed electronics, large area electronics

## Abstract

This study presents the slot-die coating process of two layers of organic materials for the fabrication of organic light emitting diodes (OLEDs). Poly(3,4-ethylenedioxythiophene) polystyrene sulfonate (PEDOT:PSS), which is commonly used in OLEDs and in organic photovoltaic devices as the hole injection layer (HIL), has been deposited via slot-die coating. Uniform films of PEDOT:PSS were obtained after optimizing the slot-die processing parameters: substrate temperature, coating speed, and ink flow rate. The film quality was examined using optical microscopy, profilometry, and atomic force microscopy. Further, poly(9,9-dioctylfluorene) (F8) and poly(9,9-dioctylfluorene-alt-benzothiadiazole) (F8BT), a well know polymer blend F8:F8BT, which is used as an emissive layer in OLEDs, has been slot-die coated. The optoelectronic properties of the slot-die coated F8:F8BT films were examined by means of photoluminescence (PL) and electroluminescence (EL) studies. The fabricated OLEDs, consisting of slot-die coated PEDOT:PSS and F8:F8BT films, were characterized to record the brightness and current efficiency.

## 1. Introduction

The manufacturing of organic electronic devices using solution-based deposition techniques, such as coating and printing, have drawn extensive attention because they can be upgraded to industrial scale [1,2,3,4,5]. From the commercial point of view these techniques have distinguishable advantages, such as the low-cost of processing and high throughput, as well as being more suitable for fabrication of flexible electronic devices in comparison to vacuum deposition techniques [6,7,8,9]. Among various printing and coating techniques that have been developed, the slot-die coating has significant advantages, due to the ability to deposit homogeneous thin films of functional materials with a high degree of reproducibility and with little waste. Many multilayer devices, including organic light emitting diodes (OLEDs) [10], photovoltaics [11], transistors [12], and sensors [13] have been fabricated by partially using this technique for the deposition of some layers. Further, the use of slot-die coating for the up-scaling of OLEDs and photovoltaics using roll-to-roll processing on flexible substrates has been successfully demonstrated [14,15]. However, the performances of devices that are partially made by engaging the solution-based techniques are often lower in comparison to devices made exclusively using vacuum-deposition-based processes. The main challenge for improving the performance of solution-processed devices is the ink formulation that will ensure homogeneous film formation and allow multi-layer deposition [16,17,18].

For display and lighting applications, OLEDs have already attracted significant commercial interest because of their unique properties such as being lightweight, high contrast, flexible, transparent, and having a wide viewing angle [19,20,21]. The simplest OLED structure consists of an emissive layer (EML) sandwiched between two electrodes: an anode and a cathode [22]. To improve the performance of the devices, additional layers are placed between the electrodes and the EML: the hole injection layer/transport layer (HIL/HTL) and electron injection layer/transport layer (EIL/ETL). These layers reduce the energy level differences between the different materials and facilitate the transfer of charge carriers into the device. In the solution-processed OLEDs, poly(3,4-ethylenedioxythiophene) polystyrene sulfonate (PEDOT:PSS) is widely used as the HIL and HTL due to its superior hole conductivity, good film formability, deep laying work function, and its ease of processing [23]. For EML fabrication, different polymeric systems are usually used because of their excellent film formability in contrast with low molecular weight materials [24,25]. One of the most commonly studied polymeric emissive layers in OLEDs is the blend of poly(9,9-dioctylfluorene-alt-benzothiadiazole) (F8BT) (acceptor and guest) and poly(9,9-dioctylfluorene) (F8) (donor and host) [26,27,28,29,30,31]. In this blend, an efficient Förster resonance energy transfer takes place, from F8 (host) to F8BT (guest), resulting in green emission with a maximum at 530 nm [32]. Previous studies on the F8:F8BT film has shown that the emissive properties of this blend are sensitive to the thermal annealing and type of solvents that are used to dissolve polymers and to deposit the film. For example, Jokinen et al. [33] reported that the thermal annealing of the F8:F8BT film at different temperatures can induce phase separation, which results in a change of the emission color, observed in both EL and in PL. They have shown that in the non-annealed films, an efficient Förster resonance energy transfer from F8 to F8BT occurs, giving the green emission typical for the F8BT. On the other hand, the film of this blend annealed at 150 °C shows an emission from both F8 and F8BT (white color) due to phase separation. Voigt et al. [34] demonstrated that the use of toluene to dissolve the F8:F8BT blend results in considerable phase separation in comparison to the blend obtained from chloroform. The authors have explained that this effect is due to lower evaporation rate of toluene during the drying process. Furthermore, literature reports have shown the possible application of polyfluorenes (F8 and F8BT) and their blend in organic light emitting transistors as well [35,36]. In all these studies, the emissive layers were formed via spin coating. However, recently slot-die coated F8BT film was used as an active layer for OLEDs, but the devices suffered from very low current efficiency at the level of only ≈0.1 cd/A [37]. It is necessary to stress that the slot-die coating of the F8:F8BT blends have not been reported on yet.

This paper is focused on the investigation of the PEDOT:PSS and F8:F8BT bilayers formed using layer-by-layer deposition via slot-die coating techniques for use in OLED devices. Major challenges in thin film formation are the control of the layer thickness and of the surface morphology, which should be smooth and uniform for device applications. The film-forming ability of the PEDOT:PSS on an indium-tin-oxide (ITO) coated glass substrate was examined by adjusting the processing parameters of the slot-die coater. After optimizing the parameters, the obtained uniform films have thicknesses ranging from 28 nm to 40 nm with an acceptable thickness variation of ±3 nm. The film of F8:F8BT was then deposited by also using the slot-die coating technique. The main test quality of F8:F8BT emissive layer was based on checking them in OLEDs. We have found that the highest brightness and current efficiency were obtained for the F8:F8BT layer with thickness of 66 ± 5 nm. The OLEDs with slot-die coated PEDOT:PSS and F8:F8BT films show homogeneous light emission with maximum brightness of 370 cd/m^2^ and a maximum current efficiency of 1.7 cd/A, which is much higher in the comparison to the previously reported results [37].

## 2. Materials and Methods

### 2.1. Materials

Poly(3,4-ethylenedioxythiophene) polystyrene sulfonate (PEDOT:PSS) dissolved in water, poly(9,9-di-n-octylfluorenyl-2,7-diyl) (F8), and poly(9,9-dioctylfluorene-alt-benzothiadiazole) (F8BT) were obtained from Ossila Ltd. (Sheffield, UK). The weight average molecular weight (M_w_) and number average molecular weight (M_n_) of F8 were 85,983 g/mol and 31,040 g/mol, respectively. The M_w_ and M_n_ of F8BT were 376,200 and 221,300 g/mol, respectively. These data were provided by the material supplier. The ITO coated glass substrates (20 Ω/▯), encapsulation glass substrates, and UV curing epoxy resin (for the OLED encapsulation) were also provided by Ossila Ltd. The solvents: acetone, isopropyl alcohol, and toluene were purchased from Sigma Aldrich (St. Louis, MO, USA) and used as provided.

### 2.2. Instruments

**Figure 1:**
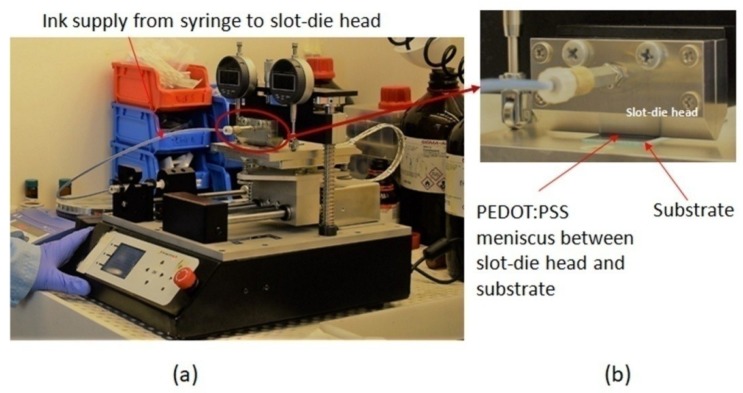
(**a**) Picture of the slot-die coater, which was placed in the fume-hood. (**b**) Image of the PEDOT:PSS meniscus formed between slot-die head and glass substrate.

Figure 1a shows the commercially available slot-die coater, designed and manufactured by Ossila Ltd. It is equipped with a high precision syringe pump (12 μm·s^−1^ to 5 mm·s^−1^) and a motorized substrate table. The table possesses a heating element, which allowed us to control the temperature from room temperature up to 120 °C. Figure 1b shows the picture of slot-die head, which had a width of 50 mm.

The fabricated OLEDs were characterized in the standard way. The current densities of the OLEDS were measured by applying voltages using an Ossila Source Measure Unit-X100, while simultaneously recording luminance using Konica Minolta LS-110 (Konica Minolta Sensing Americas, Inc., NJ, USA) luminance meter.

Absorption and emission spectra of the F8:F8BT film was measured using a Carry 5000 UV-VIS-NIR spectrometer (Varian Inc., Palo Alto, CA, USA) and FLS980 fluorescence spectrometer (Edinburgh Instruments Ltd., Livingston, UK). Thicknesses of the films were measured using a Bruker Dektak 2D profilometer (Bruker Ltd., Coventry, UK). The roughness of the samples was measured with a Flex-Axiom, Nanosurf AFM (Nanosurf GmbH, Langen, Germany).

### 2.3. Fabrication of OLEDs

**Figure 2:**
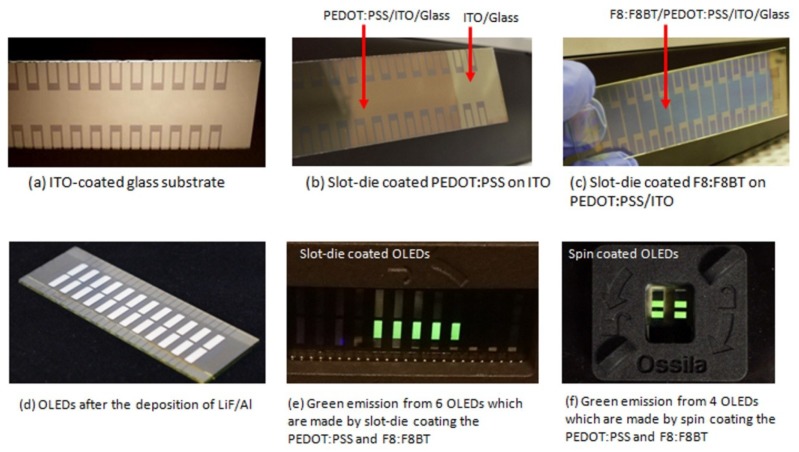
Pictures of: (**a**) indium-tin-oxide (ITO) coated glass substrates after UV-ozone cleaning, (**b**) poly(3,4-ethylenedioxythiophene) polystyrene sulfonate (PEDOT:PSS) film coated using slot-die coater, (**c**) slot-die coated poly(9,9-dioctylfluorene): poly(9,9-dioctylfluorene-alt-benzothiadiazole) (F8:F8BT) film coated on top of PEDOT:PSS layer, (**d**) 24 pixels of organic light emitting diodes (OLEDs) after the deposition of cathode, (**e**) uniform light emission from the slot-die coated OLEDs, and (**f**) uniform light emission from the spin-coated OLEDs.

Slot-die coated OLEDs were fabricated in ambient conditions. The ITO substrates for OLEDs were cleaned with hellmanex (2 mL of hellmanex III in 200 mL of hot water), acetone, and isopropanol. After cleaning, substrates were treated with UV ozone for 120 s to increase the surface energy. Immediately after surface treatment, the PEDOT:PSS solution was slot-die coated. The coated films were annealed at 150 °C for 15 min. The F8:F8BT (19:1, 15 mg/mL in toluene) solution was slot-die coated on top of the PEDOT:PSS film, and the film was annealed at 100 °C for 30 min. The F8:F8BT solution was filtered with a PTFE filter (0.45 μm pore size) prior to coating. The processing parameters of the slot-die coater used for the deposition of the PEDOT:PSS and F8:F8BT layers are listed in the Table 1. The cathode system composed of lithium fluoride (1 nm) and aluminum (100 nm) was deposited on top of the F8:F8BT film using a thermal evaporation system. After cathode deposition, all devices were encapsulated under nitrogen before their characterization. Figure 2a–d shows the images of the substrates after each step of device processing: (a) ITO coated glass substrate, (b) PEDOT:PSS film slot-die coated on top of ITO coated glass substrate, (c) F8:F8BT films slot-die coated on top of PEDOT:PSS layer, and (d) OLEDs after depositing cathode layers. Figure 2e shows the image of light emission from slot-die coated OLEDs, and Figure 2f shows the picture of the light emission from spin-coated OLEDs after the fabrication process.

In order to compare the performances of slot-die coated OLEDs and spin coated OLEDs, a batch of spin coated OLEDs was fabricated. The OLED structure was identical to those fabricated via slot-die coating. The PEDOT:PSS solution was spin-coated at 3000 rpm for 45 s, and after drying, it was covered by a spin-coated F8:F8BT layer that was deposited at 2000 rpm over 45 s. 

## 3. Results and Discussions

### 3.1. Optimization of PEDOT:PSS Slot-Die Coating

The thickness and the morphology of slot-die coated thin-film depend strongly on processing parameters. One can assume that the substrate temperature, coating speed, and dispensing rate of inks are the main parameters that influence the film morphology. Therefore, the effect of these parameters on the PEDOT:PSS film formation was investigated. After coating and drying, the film morphology was examined under an optical microscope and using profilometry.

#### 3.1.1. Influence of Substrate Temperature

The PEDOT:PSS solution was slot-die coated on ITO-coated glass substrates at different temperatures: 22 °C (RT), 40 °C, 60 °C, 80 °C, 100 °C, and 120 °C. Two other parameters: coating speed (1 mm/s) and dispensing rate (1 μL/s) were kept constant. 

**Figure 3:**
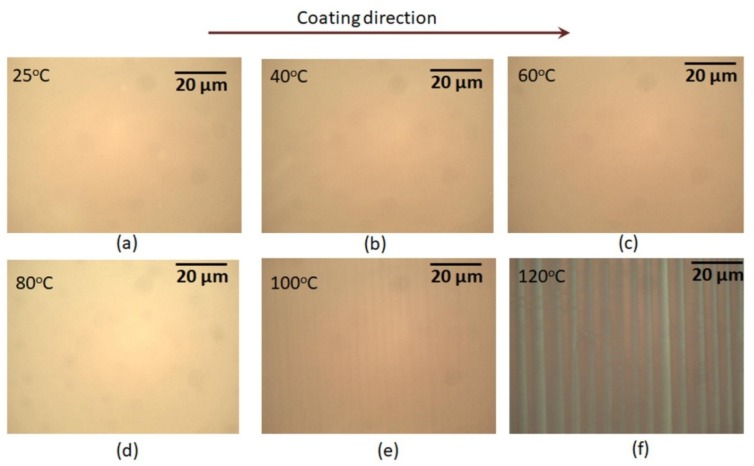
Optical image of slot-die coated PEDOT:PSS film deposited at different temperatures. The coating speed and flow rate were kept constant at 1 mm/s and 1 μL/s, respectively.

**Figure 4:**
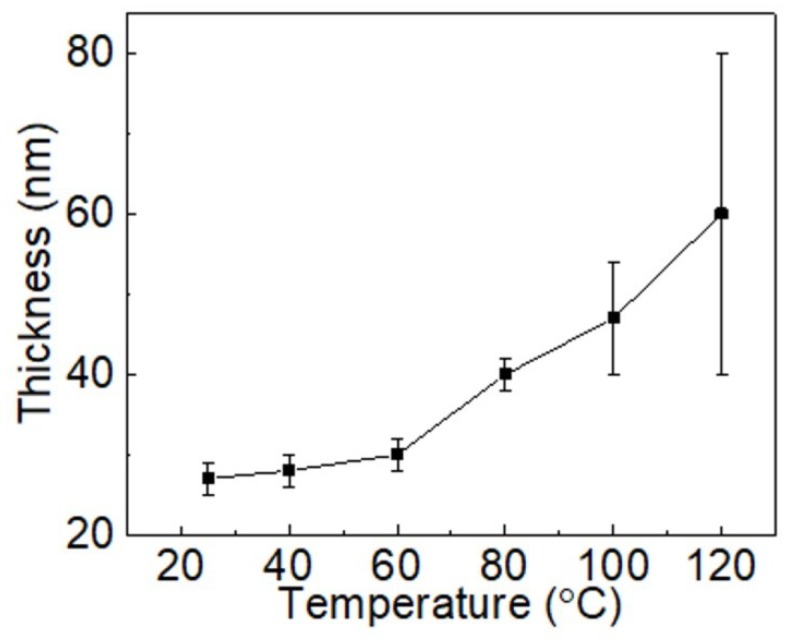
Thickness of PEDOT:PSS films that were deposited using the slot-die coating process at different temperatures. The coating speed and flow rate were kept constant at 1 mm/s and 1 μL/s, respectively. The vertical bars indicate the range of variation of the film thickness in the same sample.

At the temperatures: 22 °C (RT), 40 °C, 60 °C, and 80 °C, the formed films were smooth without any defects, as seen in Figure 3a–d. It was found that with increasing substrate temperature, the film thickness also increased. The thickness variation remained almost the same (±3 nm) for the film produced in the temperature range 22–80 °C, as shown in the Figure 4. In this study, thickness variation refers to the differences in the thickness on the same sample. However, under a further temperature increase, above 80 °C, the films were characterized using surface undulation in addition to an increase in the film thickness. Surface undulation for the films produced at temperatures 100 °C and 120 °C can be observed under an optical microscope as dark and light regular strips, as shown in Figure 3e,f. The height of the undulation was significantly higher: 30 nm at 100 °C and 42 nm at 120 °C (Figure 4). These undulation patterns possibly appeared when the evaporation rate of solvents was fast at a higher substrate temperature, which limited the material flow and precluded its uniform distribution. The boiling point of PEDOT:PSS solution is approximately 100 °C (data from manufacturer). Therefore, as the substrate reached the temperature of 100 °C or more, it was likely that solvents started to evaporate very quickly, preventing the solution from spreading uniformly. Thus, our study showed that the optimal substrate temperature is below 80 °C and it results in a uniform film of PEDOT:PSS at the coating speed of 1 mm/s and dispensing rate of 1 μm/s.

#### 3.1.2. Influence of Slot-Die Coating Speed 

The PEDOT:PSS solution was coated at six different speeds: 0.1 mm/s, 0.3 mm/s, 1 mm/s, 4 mm/s, 6 mm/s, and 10 mm/s. The substrate temperature (30 °C) and the flow rate (1 μL/s) were kept constant.

**Figure 5:**
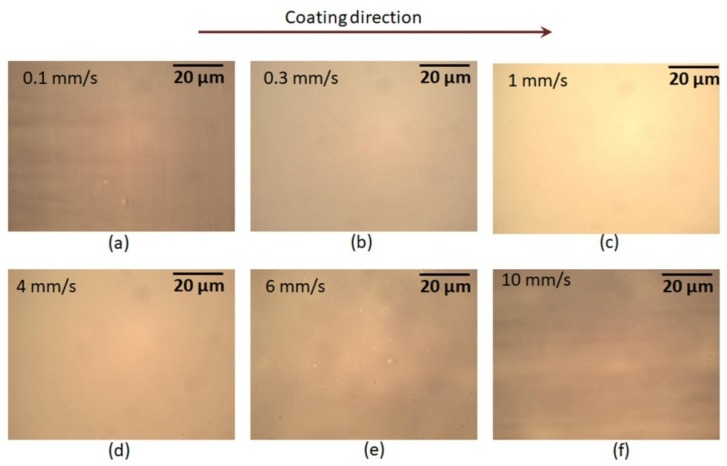
Optical images of PEDOT:PSS films that were deposited using the slot-die coating process at different coating speeds. The substrate temperature and flow rate were kept constant at 30 °C and 1 μL/s, respectively.

**Figure 6:**
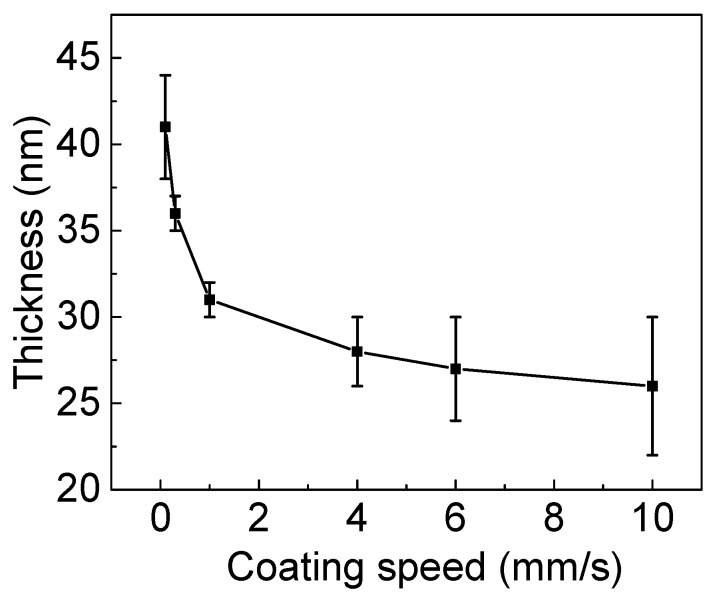
Thickness of PEDOT:PSS films that were deposited through a slot-die coating process at different coating speeds. The substrate temperature and flow rate were kept constant at 30 °C and 1 μL/s, respectively. The vertical bars indicate the range of variation of the films’ thickness in the same sample.

A low coating speed of 0.1 mm/s produced the thickest film (41 nm), which had a strip-like structure (undulation morphology) in direction orthogonal to the coating direction (Figure 5a). The height of the undulation was around 12 nm. When increasing the coating speeds to 0.3 mm/s and 1 mm/s, the films’ thicknesses decreased, producing a smooth surface without any light and dark patterns (Figure 5b,c). Additionally, the films’ thickness variations were as low as 2 nm, as seen in Figure 6. With an increase in coating speed to 4 mm/s, the film thickness further decreased. The film surface maintained smoothness without any light and dark patterns (Figure 5d). However, the variation in film thickness increased to 8 nm. At the top coating speeds of 6 mm/s and 10 mm/s, the films’ thicknesses further decreased and resulted in larger thickness variations (Figure 6). Moreover, the films’ surfaces contained tiny defects (Figure 5e,f). The obtained results suggest that the optimal coating speeds were 0.3 mm/s and 1 mm/s. The use of such slot-die coating speeds while keeping the substrate temperature at 30 °C and the flow rate at 1 μL/s resulted in defect-free uniform films.

#### 3.1.3. Influence of Ink Flow Rate

**Figure 7:**
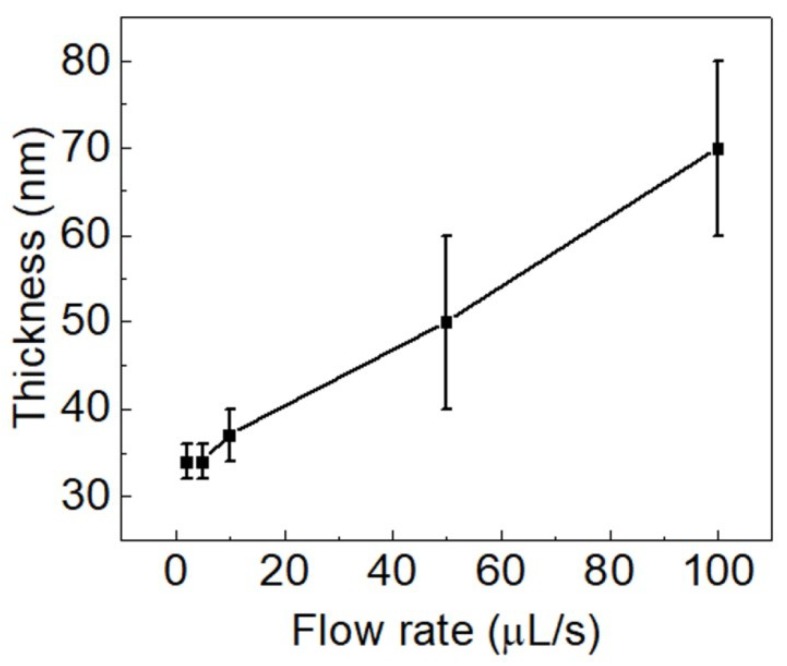
Thickness of PEDOT:PSS films which were deposited through slot-die coating process at different flow rates. The substrate temperature and coating speed were kept constant at 30 °C and 1 mm/s, respectively. The vertical bars indicate the range of variation of the film thickness in the same sample.

The solution was coated at five different flow rates: 2 μL/s, 5 μL/s, 10 μL/s, 50 μL/s, and 100 μL/s. The substrate temperature and coating speed were kept constant at 30 °C and 1 mm/s, respectively. At all flow rates, the film surface looked defect-free under an optical microscope. Films coated with flow rates of 2 μL/s and 5 μL/s had a similar thickness (34 nm) and thickness variation (2 nm), as shown in Figure 7. When increasing the flow rate to 10 μL/s, the film thickness (37 nm) and thickness variation (3 nm) increased slightly. Further increasing the flow rates to 50 μL/s and 100 μL/s, the film thickness and thickness variation increased significantly. At these flow rates (50 μL/s and 100 μL/s), it was found that the thickness of the films increased gradually from the starting point of the coating to the end of the coating. This means that the thickness was low at the beginning of the films, and that it increased gradually until the end of the film, where it was significantly higher. Such a phenomenon appears likely because at a higher flow rate, the ink is accumulating behind the slot die head and the meniscus gets gradually bigger throughout the coating. As a result, one can observe the gradual increase of the film thickness. Concluding this experimental result, the flow rate below 10 μL/s gave uniform films with an adequate thickness variation of no higher than 3 nm.

### 3.2. Slot-Die Coating of F8:F8BT Film

**Figure 8:**
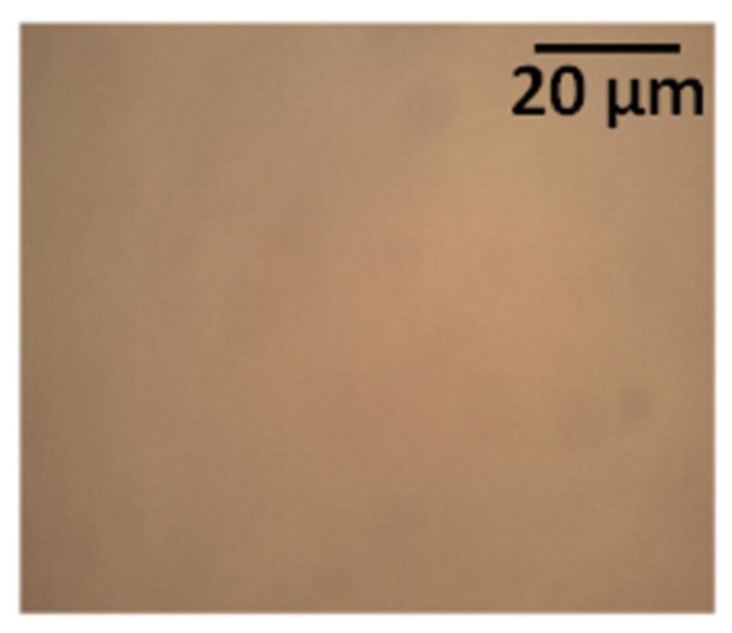
Optical image of slot-die coated F8:F8BT on a previously slot-die coated PEDOT:PSS layer.

The experiments on PEDOT:PSS described in the previous section provided information on how the differences in the slot-die coating parameters influenced the thin-film thickness and quality. Based on the knowledge gained during optimization of conditions for the slot-die coating of PEDOT:PSS films, we performed analogous procedures to optimize the processing parameters for the formation of F8:F8BT films. It was found that a substrate temperature of 30 °C, coating speed of 0.3 mm/s, and an ink flow rate of 1 μL/s resulted in a homogeneous F8:F8BT film. Such films were characterized by thicknesses of 37 ± 3 nm. However, for OLEDs application, this thickness (37 ± 3 nm) is too low. Therefore, the F8:F8BT solution was coated for a second time on the dried F8:F8BT film. Double coating resulted in a total thickness of 66 ± 5 nm, which is suitable for device operation. Optical images of the slot-die coated F8:F8BT layer is shown in Figure 8. In order to evaluate the quality of the F8:F8BT slot-die coated layers, and to optimize the coating parameters, every F8:F8BT layer was tested in OLEDs. 

### 3.3. AFM Images of Slot-Die Coated PEDOT:PSS and F8:F8BT Films

**Figure 9:**
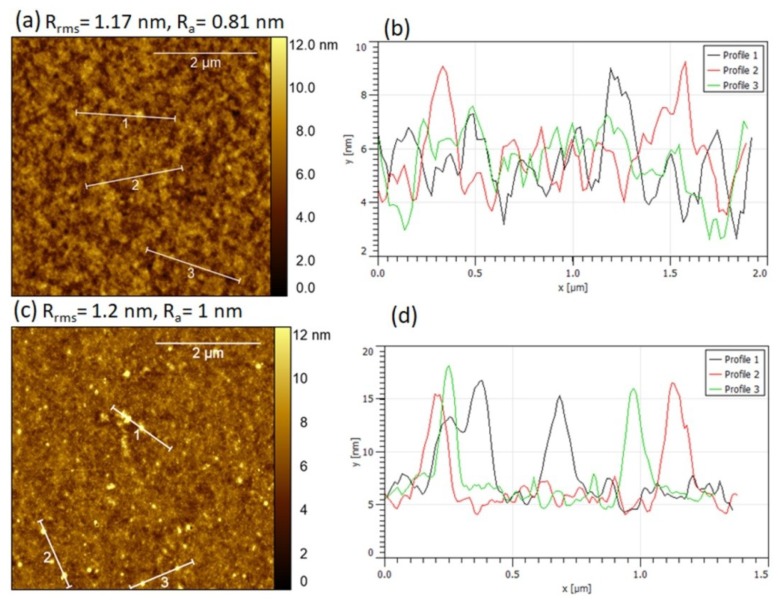
AFM height images of slot-die coated films: (**a**) PEDOT:PSS and (**c**) F8:F8BT. Surface crosses section profile of slot-die coated films: (**b**) PEDOT:PSS and (**d**) F8:F8BT. R_rms_: root mean square roughness. R_a_: roughness average.

Atomic force microscopy (AFM) was used to evaluate the morphology at the nanoscale (such as the pinholes and roughness) of the slot-die coated films. Figure 9 shows the AFM height image and surface cross section profile of the slot-die coated PEDOT:PSS and F8:F8BT film. It can be noticed that both films were free from pinholes. However, in the case of F8:F8BT, there were spikes of about 10 nm in height (Figure 9c,d), which were distributed randomly across the film. These spikes might have been formed due to aggregation of F8BT. The root mean square (R_rms_) and average roughness (R_a_) of PEDOT:PSS films over a 5 μm^2^ scan area were 1.17 nm and 0.81 nm, respectively. The root mean square (R_rms_) and average roughness (R_a_) of F8:F8BT films over a 5 μm^2^ scan area were 1.2 nm and 1 nm, respectively. Thus, a slot-die coating process was capable of producing homogeneous films with a low roughness for device applications.

### 3.4. Photoluminescence (PL) Measurements

**Figure 10:**
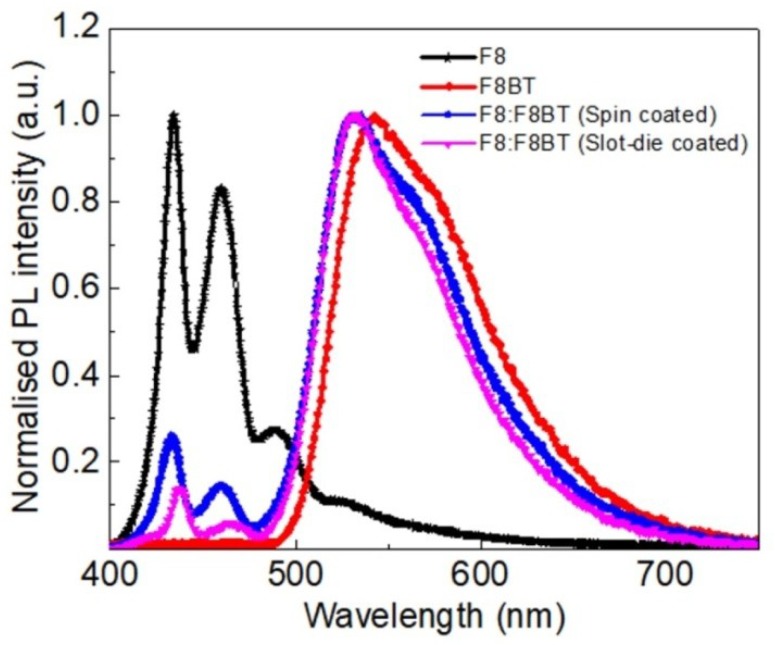
Normalized photoluminescence emission spectra of F8, F8BT films, and the film of their blend.

Figure 10 shows the normalized photoluminescence emission spectra of F8, F8BT, and their blend. Films of F8 and F8BT were formed using spin-coating, while the blended F8:F8BT films were formed using both spin-coating and slot-die coating techniques. It can be seen that the emission of the blended films was mainly dominated by F8BT, with a small contribution from F8. In addition, the PL spectra of blends were blue-shifted with respect to the spectra of the F8BT film. Such a blue shift is reported in a previous study [34]. In the F8:F8BT film, the percentage of emission from F8 was 7.4% for the spin coated film, which is similar to an earlier study [34]. The percentage of emission was calculated by comparing the emission of F8 (integrating F8:F8BT spectrum from 410 to 475 nm) with the total emission (integrating F8:F8BT spectrum from 410 nm to 700 nm). For a slot-die coated film, the percentage of emission from F8 was 3.7%, which indicated that there was an efficient Förster resonance energy transfer from F8 to F8BT. Thus, the photoluminescence emission properties of the slot-die coated blended films were similar to the properties of the films formed by the spin-coating technique. 

### 3.5. Performances of Slot-Die Coated OLEDs

**Figure 11:**
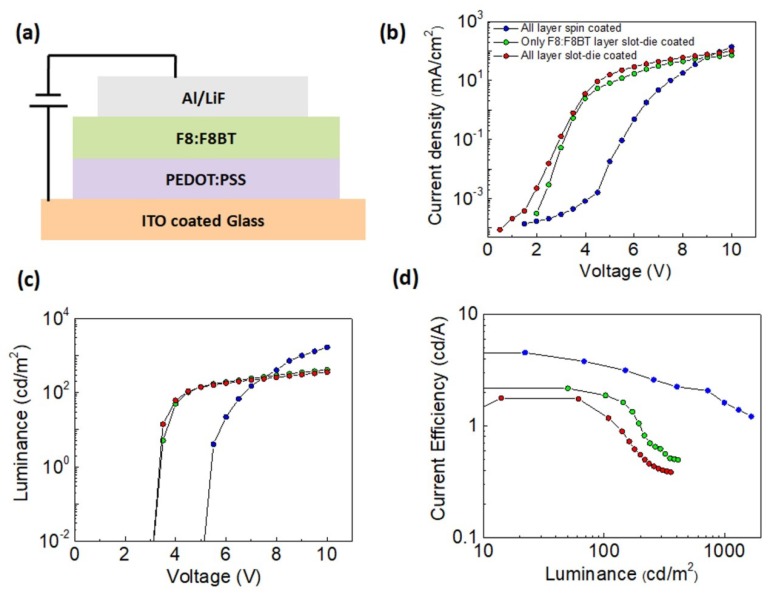
(**a**) Schematic of the OLED structure. Comparison of performances of OLEDs in which both PEDOT:PSS and F8:F8BT layers were spin-coated, both layers were slot-die coated and only F8:F8BT layers were slot-die coated: (**b**) current density vs. voltage, (**c**) luminance vs. voltage, and (**d**) current efficiency vs. luminance.

The OLEDs structure was as follows: PEDOT:PSS/F8:F8BT/LiF/Al on an ITO-coated glass substrate, which is shown in schematic form in Figure 11a. Bi-layer slot-die coated OLEDs were made by sequentially coating the PEDOT:PSS and F8:F8BT layers according to the optimized procedures, which were discussed in the earlier sections. The slot-die coating parameters are presented in Table 2. The thickness of the PEDOT:PSS layer was 34 nm with a thickness variation of ±3 nm. The deposited F8:F8BT film had a thickness of 66 nm with a thickness variation of 5 nm. For more details of the fabrication process, please refer to the experimental section.

Figure 11b–d shows the current density versus voltage (J–V), luminance versus voltage, and current efficiency of OLEDs fabricated using both slot-die coating and spin coating techniques. As expected, the spin-coated device showed a higher maximum current efficiency (4.5 cd/A) and higher brightness (1662 cd/m^2^) in comparison to the slot-die coated OLEDs. OLEDS that only had the F8:F8BT slot-die coated layer showed the maximum current efficiency of 2.1 cd/A and brightness of 411 cd/m^2^. OLEDs with all slot-die coated layers showed the maximum current efficiency of 1.7 cd/A and brightness of 357 cd/m^2^ (Table 2). The turn-on voltage of the OLEDs composed of at least the slot-die coated F8:F8BT layer was lower than for the spin-coated OLEDs. The lower turn-on voltage might be caused by a larger thickness variation in the slot-die coated layers. Such a thickness variation results in a flow of higher current in the regions of lower thickness due to the stronger electric field. Moreover, it is also possible that the thickness variation of the slot-die coated films resulted in the poor quality of the electrode and the active layer interface, bringing the device performances down. Another possible reason explaining the decrease in the performance of slot-die coated OLEDs could be the use of the low coating speed of 0.3 mm/s to coat the F8:F8BT solution, which might have led to slight etching of the previously deposited layer and mixing of the deposited compounds.

**Figure 12:**
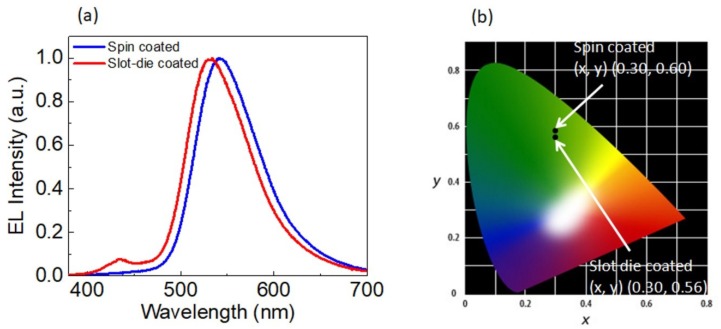
(**a**) Normalized EL and (**b**) chromaticity coordinates (CIE 1931) of spin-coated and slot-die coated OLEDs.

Figure 12a shows the EL spectra of both spin-coated and slot-die coated OLEDs. The EL spectra of a spin-coated device shows pure emission only from F8BT, as emission from F8 was completely suppressed. In contrast, the PL spectra of spin-coated films shows residual emission from F8 (Figure 10). A similar difference between EL and PL spectra have been reported by Voigt et al. [34]. The effect was explained by the fact that the excitons are mainly formed in the F8BT. In this scenario, there will be a direct recombination of electrons and holes that were residing in F8BT molecules or a recombination of electrons residing in F8BT molecules with the holes in F8 molecules. On the other hand, in the case of slot-die coated OLEDs, EL spectra show the dominant emission from F8BT with a small contribution from F8. The effect of slight differences in the EL emission between two OLEDs can also be seen in a shift of the CIE (International Commission on Illumination) color coordinates (Figure 12b). These results suggest the possibility of some de-mixing of F8 and F8BT during the film formation. This is because in the spin-coating method, the solvent evaporated quickly during spin-coating, whereas in slot-die coating, the method solvent evaporated slowly. Such a difference in evaporation rate of the solvent during film formation was likely responsible for some phase separation. Nevertheless, this effect was very weak and we can claim that there was an efficient Förster resonance energy transfer from host to guest.

It should be noted that we have not used special ink formulation for the slot-die coating process. The same solution was used for both spin-coating and slot-die coating processes. Further, the slot-die coating process involved more parameters to be optimized; for example, the distance between the slot-die coater and the substrate, and the gap between the slots. Therefore, further optimization of other process parameters and appropriate ink formulation should improve the performances of devices. Nevertheless, the performance of OLEDs demonstrates the potential applications of the slot-die coater for multi-layer coating of organic devices. 

## 4. Conclusions

We have demonstrated the possibility of the fabrication of OLEDs with two functional layers deposited layer by layer using the slot-die coating technique. It was found that the thickness and morphology of the slot-die coated films were extremely sensitive to the coating parameters such as substrate temperature, slot-die coating speed, and ink flow rates. After careful optimization of these parameters, it was possible to obtain the PEDOT:PSS layer (HIL) with an optimal thickness of 34 ± 3 nm and the F8:F8BT layer (EML) with a thickness of 66 ± 5nm. The OLEDs fabricated from these films have showed uniform light emission with a low turn-on voltage of 3.5 V, a maximum current efficiency of 1.7 cd/A, and a brightness of 357 cd/m^2^ at 10 V. The current efficiency of OLEDs obtained by us via subsequent slot-die coating of PEDOT:PSS and F8:F8BT films was almost 17 times higher compared to the results published by Raupp et al. [37] for similar OLEDs with a slot-die coated F8BT layer. It is also necessary to emphasize that the devices were fabricated by means of a simple and cheap slot-die coating machine in ambient conditions. The OLEDs performances suggest that slot-die coating is a viable deposition technique for the formation of PEDOT:PSS and F8:F8BT layers for the OLED fabrication. The observed residual emission from the host (F8) could be due to the lower drying rate of the slot-die coated films in comparison to the spin-coated films, resulting from partial phase separation in the F8:F8BT layers. We believe that through further optimization of other processing parameters, such as ink formulations, slot-die gap width, and head to substrate distance, the film uniformity and morphology can be made closer to that of spin-coated devices, resulting in improved device efficiency and brightness.

## Figures and Tables

**Table 1:** Slot-die processing parameters used for coating PEDOT:PSS and F8:F8BT films.

Slot-Die Coated Films	Substrate Temperature (°C)	Coating Speed (mm/s)	Flow Rate of Ink (μL/s)
PEDOT:PSS	60	0.5	1
F8:F8BT	35	0.3	1

**Table 2:** Thickness of PEDOT:PSS and F8:F8BT layers formed using spin-coating and slot-die coating techniques, and the performances of the OLEDs fabricated using spin-coating and slot-die coating techniques.

OLEDs Using Slot-Die Coating and/or Spin Coating Technique	PEDOT:PSS Thickness (nm)	F8:F8BT Thickness (nm)	Turn-on Voltage (V)	Luminance (cd/m^2^) at 10 V	Max. Current Efficiency (cd/A)
Slot-Die Coated OLEDs	34 ± 3	66 ± 5	3.5	357	1.77
F8:F8BT Slot-Die Coated and PEDOT:PSS Spin Coated	35 ± 1	66 ± 5	3.5	411	2.16
Spin-Coated OLEDs	35 ± 1	70 ± 1	5.5	1662	4.51

## References

[1] Riegel A.-L., Reichelt N., Scharfer P., Schabel W. (2017). Process-dependent conductivity and film homogeneity of slot-die-coated PEDOT:PSS–PVA composite films. J. Coat. Technol. Res..

[2] Raupp S.M., Merklein L., Hietzschold S., Zürn M., Scharfer P., Schabel W. (2017). Slot die-coated blue SMOLED multilayers. J. Coat. Technol. Res..

[3] Carey T., Cacovich S., Divitini G., Ren J., Mansouri A., Kim J.M., Wang C., Ducati C., Sordan R., Torrisi F. (2017). Fully inkjet-printed two-dimensional material field-effect heterojunctions for wearable and textile electronics. Nat. Commun..

[4] Ostfeld A.E., Deckman I., Gaikwad A.M., Lochner C.M., Arias A.C. (2015). Screen printed passive components for flexible power electronics. Sci. Rep..

[5] Lewis J.A., Ahn B.Y. (2015). Device fabrication: Three-dimensional printed electronics. Nature.

[6] C A., Luszczynska B., Dupont B.G.R., Sieradzki Z. (2017). Inkjet Printing Technique and Its Application in Organic Light Emitting Diodes. Disp. Imaging.

[7] Hoeng F., Denneulin A., Bras J. (2016). Use of nanocellulose in printed electronics: A review. Nanoscale.

[8] Sarma K. Recent progress in OLED and flexible displays and their potential for application to aerospace and military display systems. Proceedings of the SPIE Defense + Security.

[9] Alonso-Lomillo M.A., Dominguez-Renedo O., Arcos-Martinez M.J. (2010). Screen-printed biosensors in microbiology; a review. Talanta.

[10] Shin D., Lee J.-Y., Hong K.-Y., Park J., Seo Y.-S. (2016). Slot-die coating of organic thin films for active-matrix organic light-emitting diode displays. Thin Solid Film.

[11] Pérez-Gutiérrez E., Lozano J., Gaspar-Tánori J., Maldonado J.-L., Gómez B., López L., Amores-Tapia L.-F., Barbosa-García O., Percino M.-J. (2017). Organic solar cells all made by blade and slot–die coating techniques. Sol. Energy.

[12] Lin Z., Guo X., Zhou L., Zhang C., Chang J., Wu J., Zhang J. (2018). Solution-processed high performance organic thin film transistors enabled by roll-to-roll slot die coating technique. Org. Electron..

[13] Lee H., Lee D., Hwang J., Nam D., Byeon C., Ko S.H., Lee S. (2014). Silver nanoparticle piezoresistive sensors fabricated by roll-to-roll slot-die coating and laser direct writing. Opt. Express.

[14] Huang Y., Cha H., Chen C., Tsao C. (2017). A universal roll-to-roll slot-die coating approach towards high-efficiency organic photovoltaics. Prog. Photovolt. Res. Appl..

[15] Abbel R., de Vries I., Langen A., Kirchner G., t’Mannetje H., Gorter H., Wilson J., Groen P. (2017). Toward high volume solution based roll-to-roll processing of OLEDs. J. Mater. Res..

[16] Peters K., Raupp S., Hummel H., Bruns M., Scharfer P., Schabel W. (2016). Formation of blade and slot die coated small molecule multilayers for OLED applications studied theoretically and by XPS depth profiling. AIP Adv..

[17] Mattias A.L. (2015). Fully Slot–Die-Coated All-Organic Solar Cells. Energy Technol..

[18] Sandström A., Dam H.F., Krebs F.C., Edman L. (2012). Ambient fabrication of flexible and large-area organic light-emitting devices using slot-die coating. Nat. Commun..

[19] Volz D., Wallesch M., Flechon C., Danz M., Verma A., Navarro J.M., Zink D.M., Brase S., Baumann T. (2015). From iridium and platinum to copper and carbon: New avenues for more sustainability in organic light-emitting diodes. Green Chem..

[20] Wolfgang B., Jörg F., Tobias D.S., Bert J.S., Christian M. (2013). Device efficiency of organic light-emitting diodes: Progress by improved light outcoupling. Phys. Status Solidi A.

[21] Thejo Kalyani N., Dhoble S.J. (2012). Organic light emitting diodes: Energy saving lighting technology—A review. Renew. Sustain. Energy Rev..

[22] Chizu S., Yoshiaki T., Takeshi Y., Makoto K., Shuji D. (2014). Recent progress of high performance polymer OLED and OPV materials for organic printed electronics. Sci. Technol. Adv. Mater..

[23] Lövenich W. (2014). PEDOT-properties and applications. Polym. Sci. Ser. C.

[24] Yu D.X. (2011). Light-emitting devices with conjugated polymers. Int. J. Mol. Sci..

[25] Alsalhi M.S., Alam J., Dass L.A., Raja M. (2011). Recent advances in conjugated polymers for light emitting devices. Int. J. Mol. Sci..

[26] Morgado J., Moons E., Friend R.H., Cacialli F. (2001). De-mixing of Polyfluorene-Based Blends by Contact with Acetone: Electro- and Photo-luminescence Probes. Adv. Mater..

[27] Wilkinson C.I., Lidzey D.G., Palilis L.C., Fletcher R.B., Martin S.J., Wang X.H., Bradley D.D.C. (2001). Enhanced performance of pulse driven small area polyfluorene light emitting diodes. Appl. Phys. Lett..

[28] Morgado J., Friend R.H., Cacialli F. (2002). Improved efficiency of light-emitting diodes based on polyfluorene blends upon insertion of a poly(p-phenylene vinylene) electron- confinement layer. Appl. Phys. Lett..

[29] Wei B., Ichikawa M., Furukawa K., Koyama T., Taniguchi Y. (2005). High peak luminance of molecularly dye-doped organic light-emitting diodes under intense voltage pulses. J. Appl. Phys..

[30] Zhang Y., Blom P.W.M. (2011). Electron and hole transport in poly(fluorene-benzothiadiazole). Appl. Phys. Lett..

[31] Suh M., Bailey J., Kim S.W., Kim K., Yun D.-J., Jung Y., Hamilton I., Chander N., Wang X., Bradley D.D.C. (2015). High-Efficiency Polymer LEDs with Fast Response Times Fabricated via Selection of Electron-Injecting Conjugated Polyelectrolyte Backbone Structure. ACS Appl. Mater. Interfaces.

[32] Hill J., Heriot S.Y., Worsfold O., Richardson T.H., Fox A.M., Bradley D.D.C. (2003). Dynamics of Förster transfer in polyfluorene-based polymer blends and Langmuir–Blodgett nanostructures. Synth. Met..

[33] Jokinen K., Bykov A.V., Sliz R., Remes K., Fabritius T., Myllylä R. (2015). Light Emission Color Conversion of Polyfluorene-Blend OLEDs Induced by Thermal Annealing. IEEE Trans. Electron Devices.

[34] Voigt M., Chappell J., Rowson T., Cadby A., Geoghegan M., Jones R.A.L., Lidzey D.G. (2005). The interplay between the optical and electronic properties of light-emitting-diode applicable conjugated polymer blends and their phase-separated morphology. Org. Electron..

[35] Chuan L., Yong X., Xuying L., Takeo M., Henning S., Yong-Young N. (2015). Solution-processed high-LUMO-level polymers in n -type organic field-effect transistors: A comparative study as a semiconducting layer, dielectric layer, or charge injection layer. Semicond. Sci. Technol..

[36] Hirotake K., Takahiro O., Yutaka O. (2017). Electroluminescence emission patterns of organic light-emitting transistors based on crystallized fluorene-type polymers. Jpn. J. Appl. Phys..

[37] Raupp S.M., Merklein L., Pathak M., Scharfer P., Schabel W. (2017). An experimental study on the reproducibility of different multilayer OLED materials processed by slot die coating. Chem. Eng. Sci..

